# Mutational Signatures Are Critical for Proper Estimation of Purifying Selection Pressures in Cancer Somatic Mutation Data When Using the dN/dS Metric

**DOI:** 10.3389/fgene.2017.00074

**Published:** 2017-06-08

**Authors:** Jimmy Van den Eynden, Erik Larsson

**Affiliations:** ^1^Department of Medical Biochemistry and Cell Biology, Institute of Biomedicine, Sahlgrenska Academy, University of GothenburgGothenburg, Sweden; ^2^Unit of Public Health and Genome, Public Health and Surveillance, Scientific Institute of Public HealthBrussels, Belgium

**Keywords:** somatic mutations, cancer, mutational signatures, dN/dS, selection, purifying selection

## Abstract

Large cancer genome sequencing initiatives have led to the identification of cancer driver genes based on signals of positive selection in somatic mutation data. Additionally, the identification of purifying (negative) selection has the potential to identify essential genes that may be of therapeutic interest. The most widely used way of quantifying selection pressures in protein-coding genes is the dN/dS metric, which compares non-synonymous to synonymous substitution rates. In this study, we examine whether and how this metric is influenced by the mutational processes that have been active during tumor evolution. We use exome sequencing data from six different cancer types from The Cancer Genome Atlas (TCGA) and demonstrate that dN/dS in its basic form, where uniform base substitution probabilities are assumed, is in fact strongly biased by these mutational processes. This is particularly true in malignant melanoma, where the mutational signature is characterized by a high amount of UV-induced cytosine to thymine mutations at dipyrimidine dinucleotides. This increases the likelihood of random synonymous mutations occurring in hydrophobic amino acid codons, leading to reduced dN/dS ratios in genes encoding membrane proteins and falsely suggesting purifying selection in these genes. When this effect is corrected for by taking mutational signature-derived substitution probabilities into account, purifying selection was found to be limited and similar in all cancer types studied. Our results demonstrate that it is crucial to take mutational signatures into account when applying the dN/dS metric to cancer somatic mutation data.

## Introduction

Carcinogenesis is an evolutionary process resulting from the accumulation of somatic mutations in cancer genes (Vogelstein et al., 2013). Any mutation leading to a fitness advantage of affected cells will be positively selected for. As these driving mutations occur in driver genes, their identification is of utmost importance for the successful development of targeted cancer therapies. Therefore, different algorithms that identify signals of positive selection in somatic mutation data have been developed (Gonzalez-Perez and Lopez-Bigas, 2012; Gonzalez-Perez et al., 2013; Lawrence et al., 2013; Tamborero et al., 2013; Van den Eynden et al., 2015). In addition to positive selection, there are also indications that the genomic constitution of a tumor is further shaped by negative (or purifying) selection forces in which detrimental mutations in essential genes are selected out during tumor evolution (Lohr et al., 2012; Ostrow et al., 2014; Pyatnitskiy et al., 2015; Van den Eynden et al., 2016), although these signals appear to be less prominent.

In recent years, it has become obvious from large cancer genome initiatives like The Cancer Genome Atlas (TCGA) that the overall mutational patterns observed in tumors are also strongly influenced by heterogeneous mutational processes underlying their development, and that cancer types are characterized by different mutational signatures (Alexandrov et al., 2013; Kandoth et al., 2013; Lawrence et al., 2013). These signatures are determined by the proportion of the six main substitution classes (i.e., C>A, C>G, C>T, T>A, T>C, T>G; note that the pyrimidine of the mutated base pair is always used as a reference) and the adjacent up- and down-stream base pairs, resulting in 96 possible mutation types (6 substitution classes and 16 different combinations of up- and down-stream nucleotides).

A widely used way to quantify selection pressures in genes is the dN/dS metric (Nei and Gojobori, 1986). This metric relates the number of non-synonymous mutations per site to the number of synonymous mutations per site. Assuming the latter are not subject to any selection process, a ratio higher than 1 (i.e., more non-synonymous mutations than expected) indicates positive selection, while a ratio lower than 1 (i.e., less non-synonymous mutations than expected) indicates negative selection. Evolutionary population studies have shown that dN/dS is sensitive to assumptions about mutation probabilities (Li, 1993). Therefore, more advanced models have been suggested, taking into account differences between transition and transversion rates and codon usage bias (Goldman and Yang, 1994). Similarly, the existence of cancer-specific mutational signatures implies that the probability of a random mutation hitting a certain nucleotide depends on its sequence context, and might have an influence on the expected number of (non-)synonymous sites and hence the dN/dS metric.

Here we show that the dN/dS metric, when applied to somatic mutation data from tumors, is highly sensitive to bias introduced by mutational signatures. We show that this can give rise to false signals indicative of purifying selection, and that some gene categories are more affected than others by this effect. By incorporating these differences in mutational probabilities and using a corrected dN/dS ratio, our results indicate overall limited purifying selection in tumor evolution, with no major differences between cancer types.

## Materials and methods

### Somatic mutation data

Whole exome sequencing (WES) mutation annotation format (maf) files were downloaded from Broad Institute [Broad Institute TCGA Genome Data Analysis Center (2016): Firehose stddata__2016_01_28 run. Broad Institute of MIT and Harvard. doi: 10.7908/C11G0KM9]. Data from colon and rectal adenocarcinoma (CRC), stomach and esophageal adenocarcinoma (STES), and lung adenocarcinoma and squamous cell carcinoma (LUNG) were concatenated. Mutation data that were annotated in hg18 were converted to hg19 using UCSC's liftOver (Rosenbloom et al., 2014). All duplicate lines, identified as samples with a similar barcode and genomic location, were removed from the final dataset. Mutation annotations were determined using ANNOVAR (Wang et al., 2010). Only cancer types that contained at least 50,000 mutations in the final dataset were used for further analysis (Table 1).

**Table 1:** Summary statistics of the analyzed cancer types.

**Cancer**	**# Samples**	# Mutations (per sample^*^)	**# Genes in analysis**	dN/dS^*^	Corrected dN/dS^*^
Breast (BRCA)	980	73,242 (33)	1,278	0.91	1.04
Colorectal (CRC)	223	78,739 (93)	1,605	0.90	0.96
Lung (LUNG)	407	127,382 (245)	3,290	0.90	1.07
Malignant melanoma (SKCM)	345	241,289 (380)	6,643	0.54	0.97
Stomach and Esophageal (STES)	473	171,536 (183)	5,437	0.80	1.03
Uterus (UCEC)	247	171,230 (67)	5,360	0.95	0.99

* *median value*.

Additional somatic mutation data, called from high-coverage whole genome sequencing (WGS) data from 38 TCGA malignant melanoma samples as reported earlier (Fredriksson et al., 2014), were used for comparative analyses.

### Substitution classes

The 6 and 96 mutational substitution classes as defined by Alexandrov et al. (2013) were determined for all mutations. As the 6 substitution classes are defined as the base substitution referred to by the pyrimidine of the mutated base pair (i.e., C>A, C>G, C>T, T>A, T>C, and T>G), all purine substitutions were converted to their complementary base. For the 96 classes, additional information was used regarding the identity of the upstream and downstream base pair. Sequence information was derived from UCSC (Rosenbloom et al., 2014).

### Calculation of the basic and corrected dN/dS metric

The ratio of non-synonymous to synonymous mutations per site (i.e., dN/dS) was calculated for each gene that contained at least 10 point mutations across samples within the cancer under analysis (Nei and Gojobori, 1986):

(1) dNdS=nN sS = ns NS 

Where *n* is defined as the number of observed non-synonymous mutations (across all analyzed samples), *s* as the number of observed synonymous mutations, *N* as the number of non-synonymous positions and *S* as the number of synonymous positions.

To determine the number of (non-)synonymous positions, the three possible point mutations for each genomic position in a specific gene were simulated (i.e., each nucleotide can theoretically be mutated in three other nucleotides). The number of non-synonymous and synonymous positions was determined after annotating the simulated mutations with ANNOVAR (Wang et al., 2010).

The corrected dN/dS, defined as the observed ratio of non-synonymous to synonymous mutations normalized to the expected ratio of non-synonymous to synonymous mutations within a gene, was calculated as follows:

(2) corrected dNdS= nsNMSSMS= ns∑iNiPi∑iSiPi                                  with  i   ∈    {A[C>A]A, …,T[T>G]T}                                   (96 substitution classes)

Where *N*_*MS*_ and *S*_*MS*_ are defined as the expected number of (non-)synonymous mutations in a gene, given a prior mutational probability determined by the specific mutational process that has been operative in a specific cancer type. *N*_*i*_ and *S*_*i*_ are the number of (non-)synonymous class i substitutions per site for a given gene. *P*_*i*_ is the probability of substitution class *i*.

A one-tailed binomial test was used to check whether (corrected) dN/dS ratios were significantly lower than 1. False discovery rate corrections were done using the Benjamini–Hochberg method (Benjamini and Hochberg, 1995).

### Gene set enrichment analysis

A gene set enrichment analysis was performed to determine whether the identified genes were enriched for essential genes or specific cellular components. For the essential gene enrichment a benchmark set of human essential genes was derived from two recent CRISPR/Cas9 screens on cancer cell lines (Hart et al., 2015; Wang et al., 2015). A gene was considered essential when it was retrieved in minimal one cell line in one of both studies. These criteria yielded 5,136 essential and 13,712 non-essential genes. GO (gene ontology) gene sets were downloaded from the Molecular Signatures Database v5.0 (Subramanian et al., 2005). Enrichments were determined using Fisher's exact test.

### Protein sequence and domain data

*CCR7* amino acid sequence and domain information was downloaded from UniProt (The UniProt Consortium, 2015).

### Statistical analysis

The R statistical package was used for all data processing and statistical analysis. Details on statistical tests used are reported in the respective sections.

## Results and discussion

### Low dN/dS values in malignant melanoma do not indicate gene essentiality

Whole exome somatic mutation data from six different cancer types were downloaded from TCGA. As expected, the highest number of mutations per sample was found in malignant melanoma (median 380 mutations/sample) and lung cancer (245 mutations/sample; Table 1). Both cancers are well-known to contain a high prevalence of somatic mutations due to mutagen exposure (ultraviolet light and tobacco smoke, respectively; Vogelstein et al., 2013).

**Figure 1:**
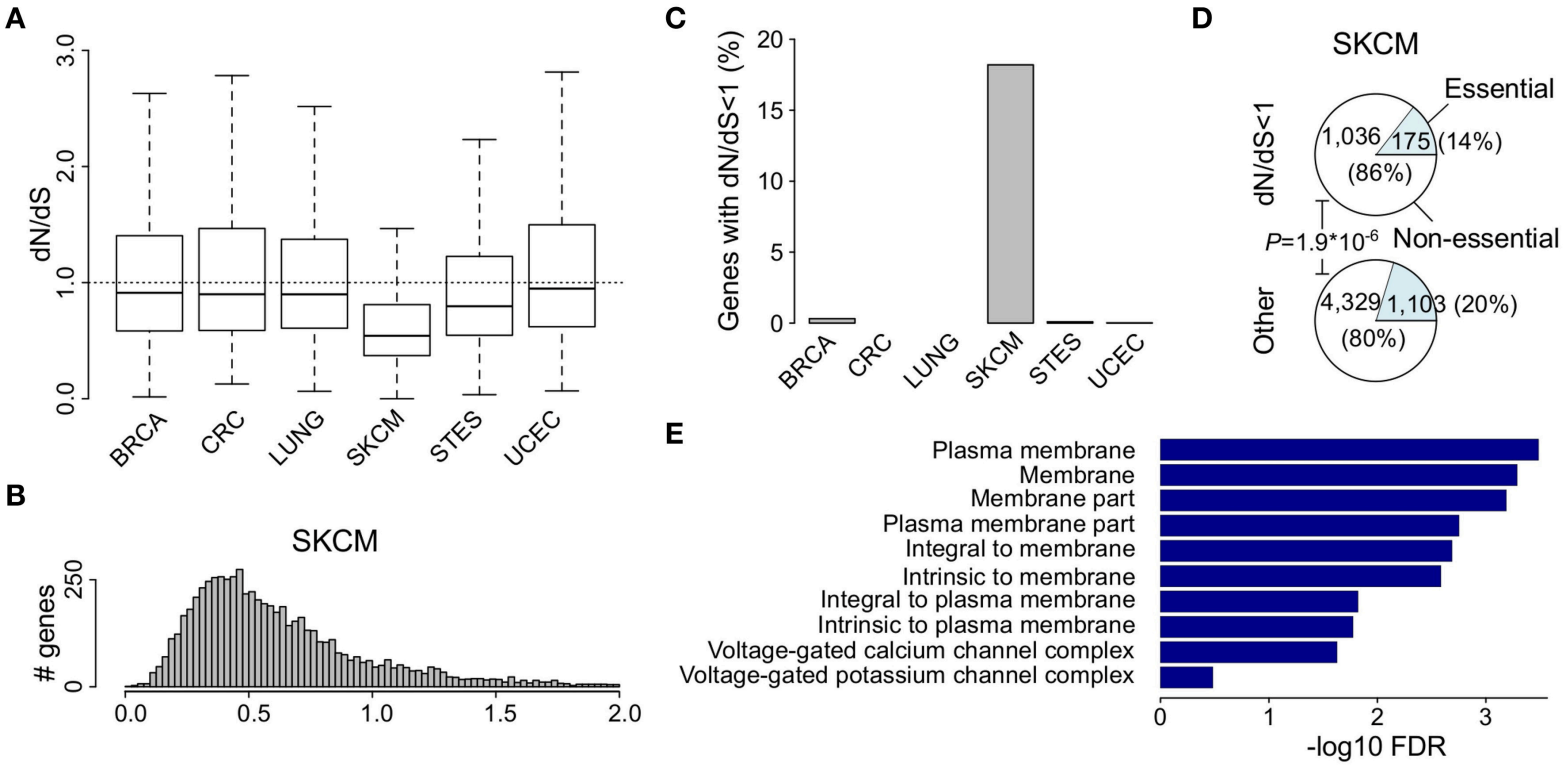
Low dN/dS values in malignant melanoma do not indicate gene essentiality. **(A)** Comparison of dN/dS values between the 6 cancer types under analysis. **(B)** Histogram of dN/dS values in malignant melanoma. **(C)** Proportion of analyzed genes that have dN/dS significantly below 1 at 5% FDR. **(D)** Pie charts show the proportion of indicated genes in malignant melanoma that are known to be essential. **(E)** GO cellular component gene set enrichment analysis for all genes that have dN/dS<1 (5% FDR) in malignant melanoma. Cellular components are ranked on FDR values as indicated and only the 10 most significantly enriched components are shown. The ratio dN/dS was calculated using a basic uniform model.

The dN/dS ratio was calculated for each gene that harbored at least 10 somatic mutations within one cancer type. The lowest dN/dS values were found for malignant melanoma (median 0.54, Figures 1A,B, Table 1, Table S1). Eighteen (1,211 out of 6,643) percent of all genes that were analyzed in this cancer had dN/dS ratios that were significantly lower than 1 (at 5% FDR), which was higher than any other cancer studied (Figure 1C). These results confirm the results from a recent study on melanoma, apparently suggesting strong purifying selective pressure acting on this cancer type (Pyatnitskiy et al., 2015). In all other cancer types, median dN/dS values were close to 1 (ranging from 0.80 to 0.95) which indicates more limited purifying selection. This is in line with results reported in breast cancer (Ostrow et al., 2014).

As purifying selection is expected to occur mainly in essential genes, we checked for enrichment of essential genes amongst the 1,211 genes with dN/dS values significantly below 1. We used a set of essential genes that were recently identified in human cancer cell lines, using the CRISPR/Cas9 technique, as a benchmark dataset (See Section Materials and Methods; Hart et al., 2015; Wang et al., 2015). Remarkably, rather than an expected enrichment, an underrepresentation of essential genes (14 vs. 20%) was found for malignant melanoma (*P* = 1.9^*^10^−6^, Fisher's exact test; Figure 1D).

To unveil which cellular processes might be underlying this apparent purifying selection, we performed a gene ontology (GO) gene set enrichment analysis (GSEA) and found strong enrichments for membrane-related activities like membrane transporters and ion channels (Table S2). Further support for this was obtained when performing the GSEA on GO cellular components only (Figure 1E and Table S2). Overall 15.2% of all 1,211 genes with dN/dS below 1 (184/1,211) are known to encode plasma membrane proteins, while this is only 10.3% for all other genes (*P* = 1.7^*^10^−6^, Fisher's exact test).

To compare the different cancer types, we repeated the GSEA for the 100 most significant genes in each cancer type. This analysis showed that the membrane enrichment was not present for any other cancer type and hence specific for malignant melanoma (Table S2).

### C to T mutations decrease the observed dN/dS values in malignant melanoma

The previous results indicate that the high number of genes with low dN/dS values that were identified in malignant melanoma are not correlated to gene essentiality and might hence not be due to purifying selection. We next checked whether differences in underlying mutational processes and hence mutational signatures might be responsible for the observed differences in dN/dS between the analyzed cancers.

**Figure 2:**
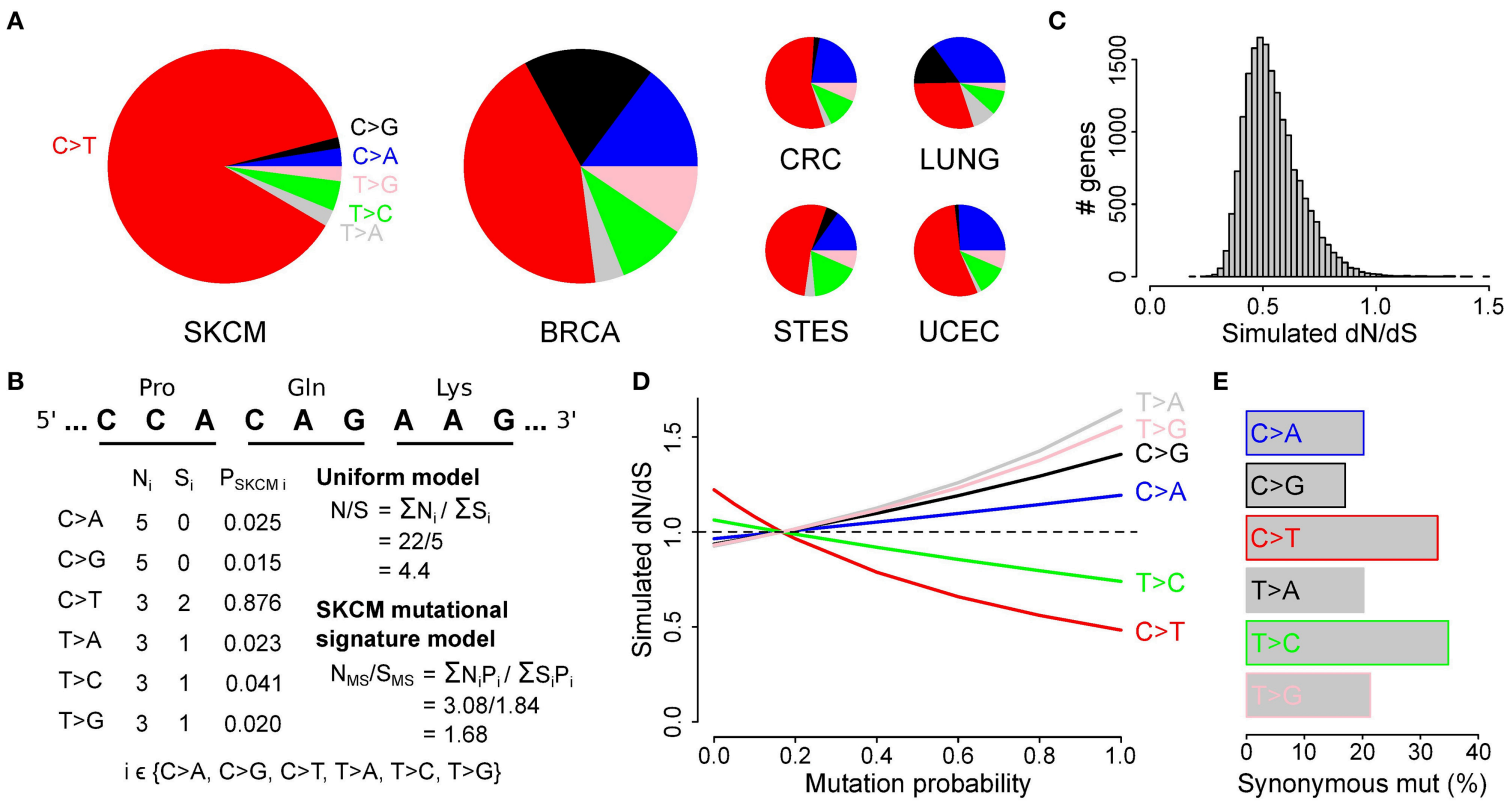
Abundant C to T mutations in malignant melanoma bias dN/dS ratios toward lower values. **(A)** Pie charts show the proportions of the 6 main substitution classes for malignant melanoma, breast cancer, and the other 4 cancers as indicated. **(B)** Calculation of the expected ratio of non-synonymous (N) to synonymous (S) mutations in 3 random exemplified codons based on the simple uniform model and after correcting for the mutation probabilities using the malignant melanoma 6-class mutational signature shown in **(A)**. **(C)** Simulation of the effect of the malignant melanoma mutation signature on the observed (uniform) dN/dS values. The simulated dN/dS values are calculated by normalizing N_*MS*_/S_*MS*_ to N/S for each gene. **(D)** Median simulated dN/dS as a function of different proportions of substitution classes (i.e., mutation probabilities) as indicated on the x-axis. **(E)** Bar plots show the proportion of all synonymous codon mutations for each substitution classes.

We first compared the proportions of the six main substitutions (i.e., C>A, C>G, C>T, T>A, T>C, T>G) between the different cancer types. While in most cancers the main substitution found was C>T, as expected, this was most pronounced in malignant melanoma with 87.6% of all somatic mutations being C>T substitutions (Figure 2A and Table S3). In lung cancer, the most prominent substitution was C>A (Figure 2A and Table S3). These results are in line with previous findings showing that polycyclic aromatic hydrocarbons in tobacco smoke cause C>A mutations in lung cancer and misrepair of UV-induced covalent bonds between dipyrimidines cause C>T mutations in malignant melanoma (Lawrence et al., 2013).

The basic calculation of dN/dS boils down to normalizing the ratio of observed non-synonymous (n) to synonymous (s) mutations to the ratio of expected non-synonymous (N) to synonymous sites (S) in a gene (Figure 2B). This uniform model assumes that at each genomic position, every mutation occurs with the same probability. As this assumption is clearly violated due to the higher mentioned differences in mutational processes, we examined whether this violation could result in an underestimation of dN/dS, possibly explaining the higher described low dN/dS values in malignant melanoma. Therefore, we first simulated the effect of the mutational processes on the uniform dN/dS ratio in malignant melanoma. Based on the proportions of the different substitution classes in malignant melanoma, any random mutation substituting a cytosine (or guanine) is expected to occur in 91.6% of all cases (2.5, 1.5, and 87.6% for C>A, C>G, and C>T, respectively; Table S3), much more frequently than the 8.4% of mutations hitting a thymine (or adenine; 2.3, 4.1, and 2.0% for T>A, T>C, and T>G, respectively; Table S3). By incorporating these six mutation probabilities in the calculation of the N/S ratio, as exemplified by N_*MS*_/S_*MS*_ in Figure 2B, and normalizing this to the uncorrected N/S ratio, we simulated the effect of the specific melanoma mutational signature on the (uniform) dN/dS values of 17,437 different genes. This simulation resulted in a surprisingly high amount (17,361) of genes having simulated dN/dS values below 1 and hence a clear underestimation of dN/dS (Figure 2C and Table S4). This effect was present for all cancers, but was most pronounced for malignant melanoma (Figure S1A).

To further investigate the effect of different mutation probabilities on the dN/dS metric, we used a similar simulation approach by changing the probability of one substitution class and keeping the probability of the other classes constant (Figure 2D). The results show a clear drop in dN/dS when the probability of C>T, or to a lesser extent T>C, was increased. As the redundancy of the genetic code is most prominent for C>T (or G>A at the other strand) interchanges at the 3^d^ nucleotide positions, this decrease in dN/dS is most likely caused by the higher occurrence of synonymous mutations for C>T and T>C substitutions as compared to the other substitution classes (Figure 2E).

The results from these simulations suggest that the low dN/dS values in malignant melanoma are not due to purifying selection, but rather to a C>T mutational signature effect. This also explains why genes with low dN/dS values are not enriched for essential genes. However, this bias toward more synonymous mutations could not explain the earlier described enrichment of membrane proteins (Table S5).

### Upstream thymine residues increase the number of mutations hitting genes coding for membrane proteins

**Figure 3:**
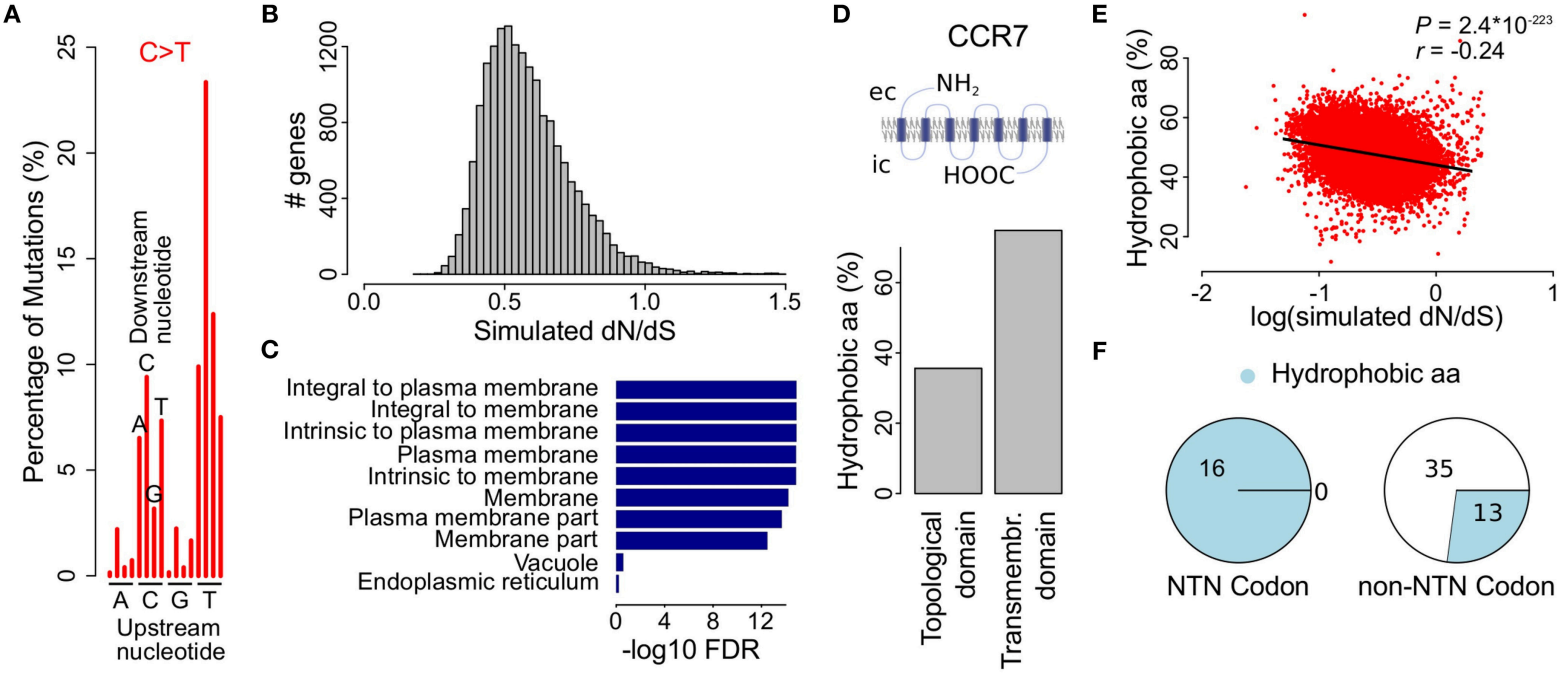
Simulations using the melanoma 96-class signature show decreased dN/dS values in genes coding for membrane proteins. **(A)** Bars indicate different proportions of up- and downstream nucleotides for C>T substitutions in malignant melanoma. See Figure S2 for the complete 96-class mutational signature. **(B)** Simulation of the effect of the malignant melanoma 96-class mutational signature on the observed dN/dS values. **(C)** GO cellular component gene set enrichment analysis for genes with the lowest simulated dN/dS values (i.e., below 0.35). Cellular components are ranked on significance (bars) and only the 10 most significantly enriched components are shown. **(D)** Membrane proteins are composed of transmembrane and topological domains. This structure is exemplified by *CCR7*, a gene that codes for a G-protein coupled receptor and was found to have low simulated dN/dS values. Bar plot compares the proportion of hydrophobic amino acids between the topological and the transmembrane domain. ec, extracellular; ic, intracellular. **(E)** Scatter plot shows the percentage of hydrophobic amino acids as a function of the simulated dN/dS value (log scale). Pearson correlation *P*- and *r*-values are indicated on top. **(F)** Pie charts show the distribution of hydrophobic amino acids in codons with or without a thymine on the second nucleotide position.

As it has been shown that, apart from the six main substitutions, the mutational processes at play in a given cancer are more accurately described by taking the adjacent bases into account (Alexandrov et al., 2013), we next determined the proportion of the resulting 96 substitution classes for each cancer. As expected the most pronounced substitution class in malignant melanoma was TCN>TTN (53.1%) and more specific TCC>TTC (23.3%), i.e., a C>T substitution in which the upstream nucleotide is a T and the downstream nucleotide is a C (Figure 3A, Figure S2 and Table S3).

We used the mutational probabilities derived from these 96 substitution classes to simulate their effect on the observed dN/dS ratio, using the approach described higher. This resulted again in a clear downward shift of dN/dS, with 98.2% (17,124 out of 17,437) of all genes having simulated dN/dS values below 1 (Figure 3B). While these results are comparable to the six-substitution class probability model (Figure 2C), a gene set enrichment analysis now did result in a strong enrichment of membrane proteins (Figure 3C and Table S5), like what we described earlier in Figure 1E for the observed dN/dS values in melanoma. The simulated dN/dS differences between the cancer types were also more similar to the observed dN/dS ratios when the 96-class model was used as compared to the 6-class model (Figure S1).

Membrane proteins are composed of one or more transmembrane domains, parts of the protein that are in direct contact with the hydrophobic phospholipid bilayer of the cellular membrane. The formation of a stable interaction with the membrane implies an abundance of hydrophobic amino acids (i.e., ala, gly, ile, leu, phe, val, pro, met, and trp) in this part of the protein, as exemplified in Figure 3D by *CCR7*, one of the genes with the lowest melanoma 96-class simulated dN/dS values (0.25, Table S4). Therefore, we hypothesized that the melanoma 96-class signature not only leads to a higher probability of synonymous mutations, as shown higher, but more specifically of synonymous mutations in hydrophobic amino acid codons, explaining the enrichment of membrane proteins. We could indeed demonstrate a higher proportion of mutations hitting hydrophobic amino acid codons in the genes having the lowest 96-class simulated dN/dS values as compared to the genes having higher simulated dN/dS values (Pearson correlation *P* = 2.4^*^10^−223^; Figure 3E). This enrichment was found to be attributed to the high frequency (16/16) of NTN codons (i.e., codons with a T on their second position) that code for hydrophobic amino acids as compared to other codons (13/48; Figure 3F). A high frequency of TpC mutations in melanoma, together with the fact that synonymous mutations occur in the third position of a codon, explains why synonymous substitutions are particularly common in these amino acids.

### Corrected dN/dS values suggest limited purifying selection in cancer

As it is obvious from the previous results that differences in mutational signatures have a strong effect on the observed dN/dS ratios when using a uniform model, this parameter should be used with care when examining selection processes in cancer somatic mutation data. This is illustrated by the striking resemblance between the simulation results from this study and the earlier reported findings of purifying selection in genes coding for membrane proteins in melanoma based on the (uniform) dN/dS metric (Pyatnitskiy et al., 2015).

**Figure 4:**
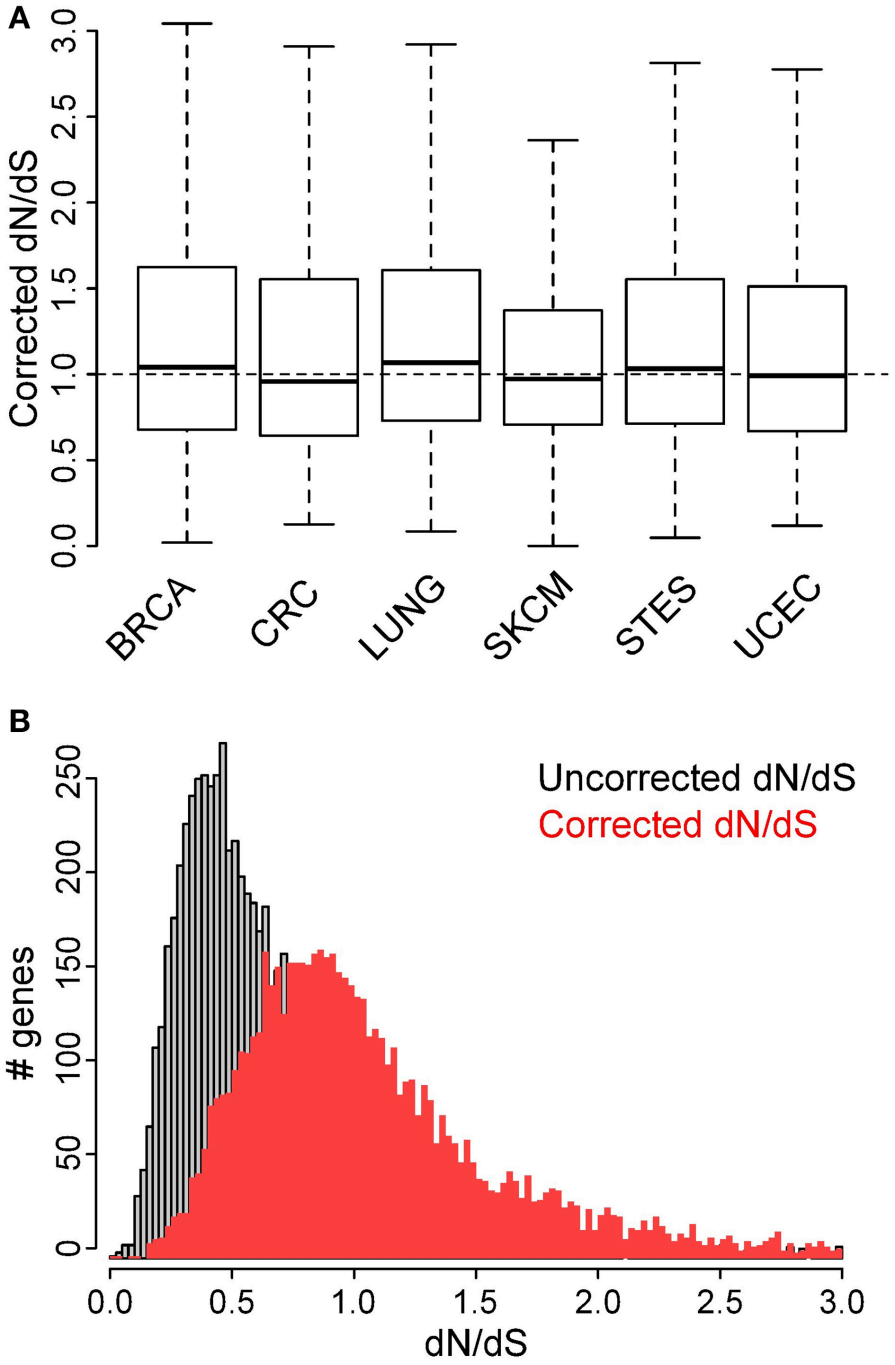
Corrected dN/dS ratios, calculated using the 96-class mutation probabilities, do not indicate major differences between cancer types. **(A)** Comparison of dN/dS values between the 6 cancer types under analysis. **(B)** Comparison of histograms for uncorrected (gray) and corrected (red) dN/dS values for all genes that contain at least 10 point mutations in malignant melanoma.

Therefore, we suggest using a corrected dN/dS metric in which N and S do not represent the number of (non-)synonymous sites but the expected number of (non-)synonymous mutations at these sites, given the probabilities derived from the specific mutational signature. These corrected dN/dS values were found to be higher (median 0.97 vs. 0.54) than the uncorrected dN/dS values in malignant melanoma (Figure 4 and Table 1), and no major differences were observed between cancer types anymore. Finally, no membrane protein enrichments were found anymore for the genes with the lowest corrected dN/dS values (Table S6), confirming that there is no actual purifying selection of genes coding for membrane proteins.

These results suggest that purifying selection is overall rather limited in cancer and not different between cancer types. A potential issue is the intrinsic assumption that selection processes do not have a major influence on the observed mutational signature in a cancer type itself. While this assumption seems solid for positive selection, where the majority of mutations have been shown to be passenger events (Vogelstein et al., 2013), it might be less trivial for purifying selection. Related to this, the dN/dS correction was done on the same data that were used to derive the mutation probabilities used for the correction. To solve both issues we recalculated the corrected dN/dS values using mutation probabilities derived from a small set of WGS data from 38 malignant melanoma samples, containing a total number of 3,596,899 somatic mutations (Fredriksson et al., 2014). We observed similar signatures between WGS and WES data and between exonic (containing 33,294 somatic mutations) and non-exonic (containing 3,563,605 somatic mutations) genomic regions of the WGS data (Figure S3A). As expected, this resulted in similar (corrected) dN/dS distributions when mutation probabilities were derived from the exonic or non-exonic regions of WGS data or from WES data (Figure S3B). These findings suggest the validity of the correction approach on an independent dataset and do not suggest any effect of selection on the mutational signature itself, which would lead to larger differences between exonic and non-exonic mutational signatures.

Because a minimal amount of somatic mutations is required for dN/dS to be reliably calculable within a gene, our analysis was restricted to genes containing a minimal number of somatic mutations across samples. This implies certain genes under purifying selection might be excluded from analysis, possibly leading to an overestimation of dN/dS when comparing cancers in Figure 4A. Future analyses on larger datasets, were sufficient somatic mutations are present for all genes to be analyzable within each cancer, are required to explore this further and to add additional cancer types to the analysis. It is important to realize however that the simulations in Figures 2, 3 were all performed on a complete set of genes in all cancers analyzed. Furthermore, the large increase in dN/dS values in malignant melanoma after correction (Figure 4B) cannot be explained by any filtering bias.

## Conclusion

In this study, we have shown that differences in mutational processes that have been active during tumor evolution can have a large impact on the expected number of synonymous and non-synonymous mutations in a gene. While this is a global analysis, where subclonality and intratumoral heterogeneity have not been taken into account, it is clear that not considering the resulting differences in mutational signatures might lead to false conclusions regarding selection pressures as quantified using the dN/dS metric. In conclusion, it is critical that mutational signatures are taken into account when calculating dN/dS values based on somatic mutation data from tumors.

## Author contributions

JV and EL designed the study. JV was responsible for data analysis and drafted the manuscript. Both authors discussed the results and finalized the manuscript.

### Conflict of interest statement

The authors declare that the research was conducted in the absence of any commercial or financial relationships that could be construed as a potential conflict of interest.

## Supplementary material

Table S1 dN/dS ratios in 6 cancer types. The simple, 6-class corrected and 96-class corrected dN/dS metric was calculated for each gene that harbored at least 10 mutations. *P*-values are calculated using a one-tailed binomial test. *Q*-values are calculated using the Benjamini–Hochberg method. Cancer types are indicated by the tab names.

Table S2 GO gene set enrichment results on genes with low observed dN/dS values. A gene set enrichment analysis for GO and GO cellular components was performed on all genes that had dN/dS below 1 at 5% FDR in malignant melanoma (SKCM tab) and for the 100 genes with lowest *p*-values in each cancer type (indicated by tab names). GO terms are ranked based on increasing enrichment *p*-values.

Table S3 6-class and 96-class mutational signatures for all cancer types. For each cancer type (row names) the proportions of the different substitution classes (column names) are given.

Table S4 Simulated dN/dS values in 6 cancer types. 6-class (first tab) and 96-class (second tab) mutational signature effect on dN/dS was simulated in 17,437 genes. The simulated dN/dS values were calculated by normalizing N_*MS*6_/S_*MS*6_ (or N_*MS*96_/S_*MS*96_) to N/S for each gene as indicated in Figure 2B.

Table S5 GO cellular components gene set enrichment results on genes with low simulated dN/dS values. A gene set enrichment analysis for GO cellular components was performed for the genes that had simulated dN/dS values below 0.35. Simulations were performed using the 6-class and the 96-class malignant melanoma signature, respectively (indicated by tab names). Cellular components are ranked based on increasing enrichment *p*-values.

Table S6 GO cellular components gene set enrichment results for genes with low observed corrected dN/dS values. A gene set enrichment analysis for GO cellular components was performed on each cancer type (indicated by tab names) for the 100 genes with lowest *p*-values. Cellular components are ranked based on increasing enrichment *p*-values.

Figure S1 Comparison of simulated dN/dS values between cancer types. The ratio dN/dS was simulated based on mutation probabilities derived from the 6-substitution **(A)** or 96-substitution **(B)** class model.

Figure S2 96-class mutational signatures for different cancer types. Bars indicate the percentage of each of 96 mutation types, determined by the substitution class and the adjacent up- and downstream nucleotide. Cancer type is indicated above every chart.

Figure S3 Comparison of mutational signatures derived from whole exome- (WES) and whole genome sequencing (WGS) data in malignant melanoma. **(A)** Comparison of the 96-class mutational signatures derived from 345 WES malignant melanoma samples (upper panel), the exonic (middle panel), and non-exonic (lower panel) genomic regions from 38 WGS malignant melanoma samples. Bars indicate the percentage of each of 96 mutation types, determined by the substitution class and the adjacent up- and downstream nucleotide. **(B)** Comparison of dN/dS values after correction using mutational probabilities derived from different data sources as indicated.
