# Supplementary material for: Mutational Signatures Are Critical for Proper Estimation of Purifying Selection Pressures in Cancer Somatic Mutation Data When Using the dN/dS Metric
Source: Front Genet. 2017 Jun 8;8:74. doi: 10.3389/fgene.2017.00074 (PMC5462936; doi:10.3389/fgene.2017.00074)
Supplement: Figure S1 — Comparison of simulated dN/dS values between cancer types. The ratio dN/dS was simulated based on mutation probabilities derived from the 6-substitution (A) or 96-substitution (B) class model. [file DataSheet1.PDF]

Supplementary figures

A

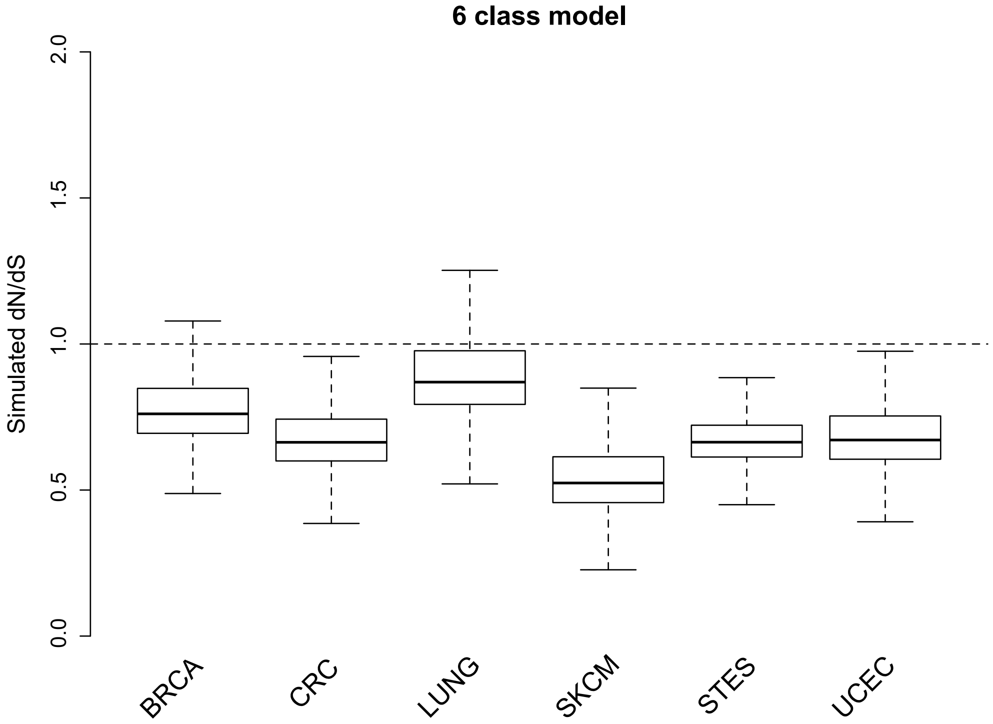

B

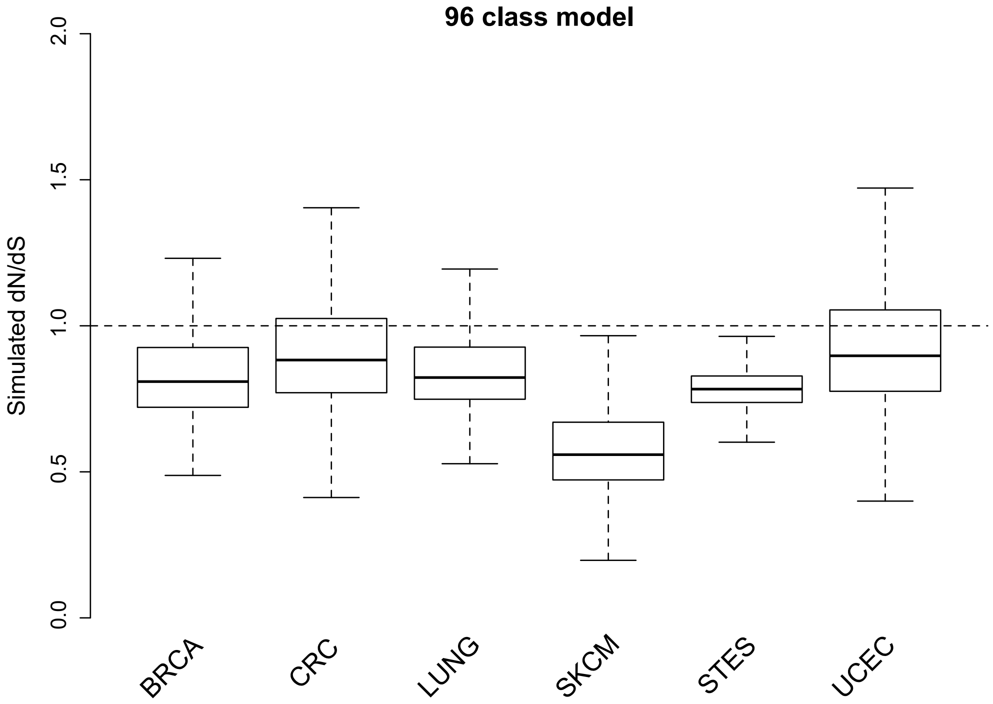

S1 Figure. Comparison of simulated dN/dS values between cancer types. The ratio dN/dS was simulated based on mutation probabilities derived from the 6-substitution (A) or 96-substitution (B) class model.

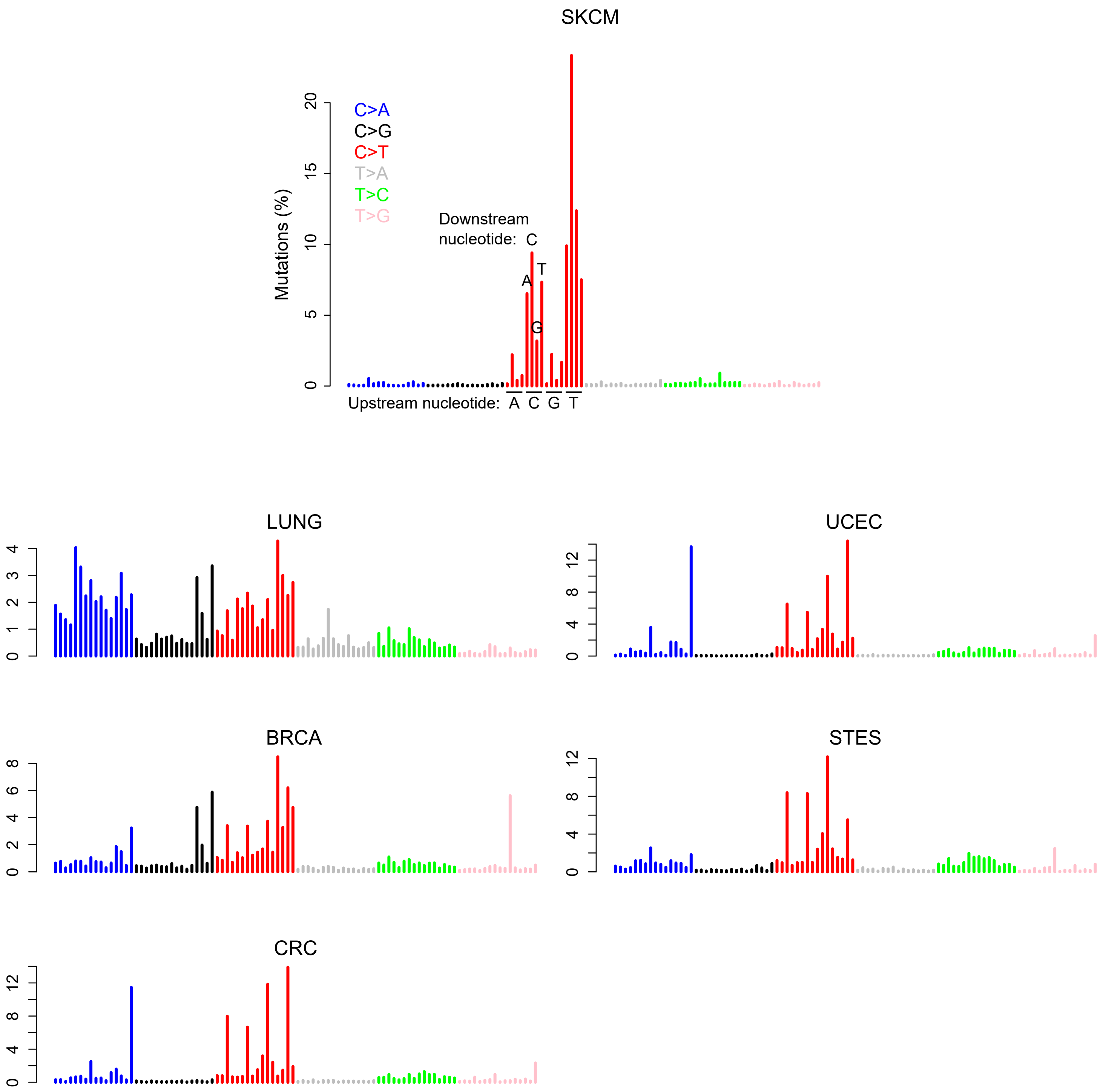

S2 Figure. 96-class mutational signatures for different cancer types. Bars indicate the percentage of each of 96 mutation types, determined by the substitution class and the adjacent up- and downstream nucleotide. Cancer type is indicated above every chart.

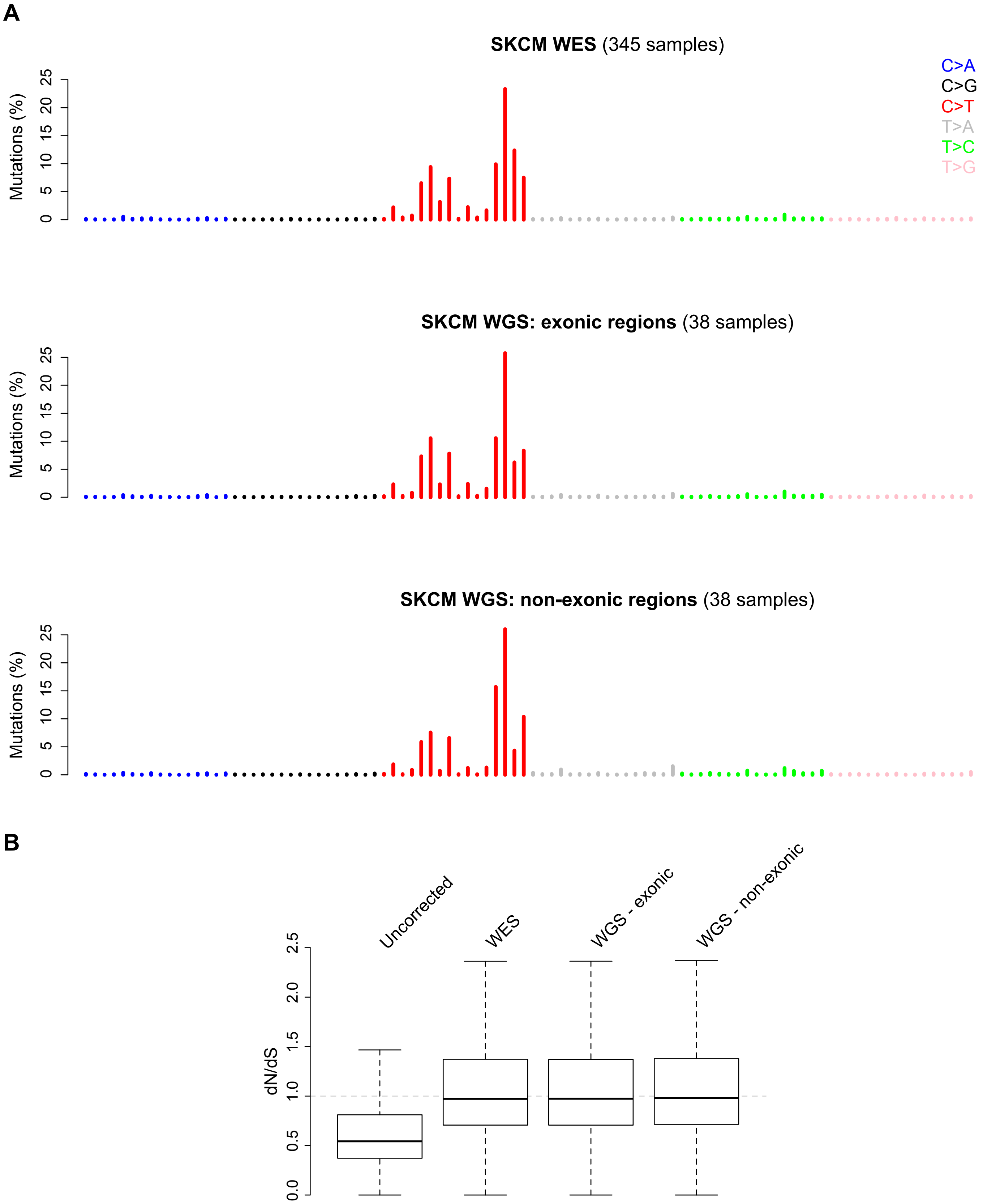

S3 Figure. Comparison of mutational signatures derived from whole exome- (WES) and whole genome sequencing (WGS) data in malignant melanoma. A) Comparison of the 96-class mutational signatures derived from 345 WES malignant melanoma samples (upper panel), the exonic (middle panel) and non-exonic (lower panel) genomic regions from 38 WGS malignant melanoma samples. Bars indicate the percentage of each of 96 mutation types, determined by the substitution class and the adjacent up- and downstream nucleotide. B) Comparison of dN/dS values after correction using mutational probabilities derived from different data sources as indicated.
